# The impact of FGF19/FGFR4 signaling inhibition in antitumor activity of multi-kinase inhibitors in hepatocellular carcinoma

**DOI:** 10.1038/s41598-021-84117-9

**Published:** 2021-03-05

**Authors:** Hiroaki Kanzaki, Tetsuhiro Chiba, Junjie Ao, Keisuke Koroki, Kengo Kanayama, Susumu Maruta, Takahiro Maeda, Yuko Kusakabe, Kazufumi Kobayashi, Naoya Kanogawa, Soichiro Kiyono, Masato Nakamura, Takayuki Kondo, Tomoko Saito, Ryo Nakagawa, Sadahisa Ogasawara, Eiichiro Suzuki, Yoshihiko Ooka, Ryosuke Muroyama, Shingo Nakamoto, Shin Yasui, Akinobu Tawada, Makoto Arai, Tatsuo Kanda, Hitoshi Maruyama, Naoya Mimura, Jun Kato, Yoh Zen, Masayuki Ohtsuka, Atsushi Iwama, Naoya Kato

**Affiliations:** 1 Department of Gastroenterology, Graduate School of Medicine, Chiba University, 1-8-1 Inohana, Chuo-ku, Chiba, 260-8670 Japan; 2 Department of Molecular Virology, Graduate School of Medicine, Chiba University, 1-8-1 Inohana, Chuo-ku, Chiba, 260-8670 Japan; 3 Department of Gastroenterology and Hepatology, Nihon University School of Medicine, 30-1 Oyaguchi-Kamicho, Itabashi-ku, Tokyo, 173-8610 Japan; 4 Department of Gastroenterology, Juntendo University School of Medicine, 2-1-1 Hongo, Bunkyo-ku, Tokyo, 113-8421 Japan; 5 Department of Transfusion Medicine and Cell Therapy, Chiba University Hospital, 1-8-1 Inohana, Chuo-ku, Chiba, 260-8670 Japan; 6 Institute of Liver Studies, King’s College Hospital, London, UK; 7 Department of General Surgery, Graduate School of Medicine, Chiba University, 1-8-1 Inohana, Chuo-ku, Chiba, 260-8670 Japan; 8 Division of Stem Cell and Molecular Medicine, Center for Stem Cell Biology and Regenerative Medicine, The Institute of Medical Science, The University of Tokyo, 4-6-1 Shirokanedai, Minato-ku, Tokyo, 108-8639 Japan

**Keywords:** Cancer, Molecular biology, Biomarkers, Gastroenterology

## Abstract

FGF19/FGFR4 autocrine signaling is one of the main targets for multi-kinase inhibitors (MKIs). However, the molecular mechanisms underlying FGF19/FGFR4 signaling in the antitumor effects to MKIs in hepatocellular carcinoma (HCC) remain unclear. In this study, the impact of FGFR4/ERK signaling inhibition on HCC following MKI treatment was analyzed in vitro and in vivo assays. Serum FGF19 in HCC patients treated using MKIs, such as sorafenib (n = 173) and lenvatinib (n = 40), was measured by enzyme-linked immunosorbent assay. Lenvatinib strongly inhibited the phosphorylation of FRS2 and ERK, the downstream signaling molecules of FGFR4, compared with sorafenib and regorafenib. Additional use of a selective FGFR4 inhibitor with sorafenib further suppressed FGFR4/ERK signaling and synergistically inhibited HCC cell growth in culture and xenograft subcutaneous tumors. Although serum FGF19^high^ (n = 68) patients treated using sorafenib exhibited a significantly shorter progression-free survival and overall survival than FGF19^low^ (n = 105) patients, there were no significant differences between FGF19^high^ (n = 21) and FGF19^low^ (n = 19) patients treated using lenvatinib. In conclusion, robust inhibition of FGF19/FGFR4 is of importance for the exertion of antitumor effects of MKIs. Serum FGF19 levels may function as a predictive marker for drug response and survival in HCC patients treated using sorafenib.

## Introduction

Hepatocellular carcinoma (HCC) is the fourth largest cause of cancer death globally^1^. Risk factors for HCC are chronic hepatitis (CH) and liver cirrhosis (LC). They are followed by chronic hepatitis B virus (HBV) and hepatitis C virus (HCV) infection, alcohol consumption and metabolic syndrome^2^. The number of new HCC cases has been gradually increasing^3^. However, many HCC patients are diagnosed at advanced stages and their long-term survival remains poor, with a 5-year survival rate of less than 20%^4,5^.

Advances in next-generation sequencing (NGS) technology revealed genetic alterations, such as mutation, copy number variation and HBV integration, in HCC tissues^6,7^. However, such alterations are not necessarily frequent, and druggable mutations have been identified in solid tumors, such as lung cancer and breast cancer, but not in HCC. Multi-kinase inhibitors (MKIs), such as sorafenib, regorafenib, and lenvatinib, which inhibit multiple tyrosine kinase type receptors, are generally used to treat advanced HCC, although the survival benefits are limited^8–10^. Since the pharmacodynamic properties of the MKIs are quite different (Supplementary Table S1)^11,12^, understanding the molecular mechanisms of MKI activity is essential for both drug selection and prognosis prediction.

Fibroblast growth factor 19 (FGF19), a secreted protein from the small intestine, has unique specificity for FGF receptor 4 (FGFR4)^13^. FGF19/FGFR4 signaling regulates many biological processes such as cellular proliferation, differentiation, angiogenesis, and the metabolism of bile acid, glucose and lipids^14–17^. Of note, autocrine secretion of FGF19 has been reported in a variety of cancers, including HCC^18^. Although *FGF19* transgenic mice develop HCC, crossing with *FGFR4* knockout mice rescues the phenotype^19,20^. Together, these findings suggest the importance of FGF19/FGFR4 signal regulation in HCC. Although FGFR4 is one of the main targets of MKIs, the role of FGF19/FGFR4 signaling inhibition remains to be elucidated.

In this study, we examined the contribution of FGF19/FGFR4 signaling to the antitumor effects in culture and in an in vivo transplant model. To evaluate the expression of FGF19 and FGFR4, clinicopathological analyses using HCC surgical specimens were conducted. We also investigated whether serum FGF19 levels can function as a predictive marker for treatment response of MKIs using enzyme-linked immunosorbent assay (ELISA).

## Results

### Basal expression of FGF19/FGFR4 in HCC cells

**Figure 1:**
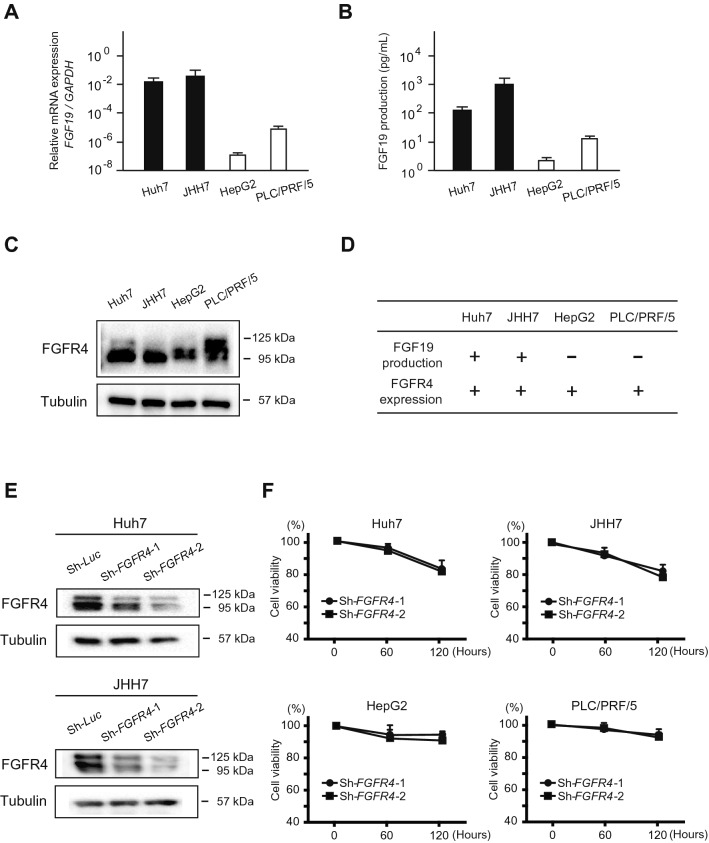
Basal expression of FGF19/ FGFR4 and *FGFR4*-knockdown in HCC cells. (**A**) FGF19 mRNA expression in HCC cells. (**B**) FGF19 secretion in HCC cells. (**C**) FGFR4 protein levels in HCC cells. (**D**) Summary of the FGF19/FGFR4 expression pattern in HCC cells. (**E**) Cells transduced with the indicated lentiviruses were subjected to Western blot analyses using anti-FGFR4 antibody and anti-tubulin antibody (loading control). (**F**) Cell growth inhibition in *FGFR4*-knockdown HCC cells 60 and 120 h after seeding. These data were from three independent experiments.

To investigate the role of FGF19/FGFR4 signaling, we first evaluated the expression of FGF19 and FGFR4 in human HCC cell lines, namely Huh7, JHH7, HepG2 and PLC/PRF/5 cells. Of note, FGF19 mRNA expression varied among these cells and was consistent with FGF19 production in the culture supernatant (Fig. 1A,B). FGF19 mRNA expression in Huh7 and JHH7 cells and the FGF19 concentration in supernatants of these cells were significantly higher than those in HepG2 and PLC/PRF/5 cells. Subsequent Western blotting demonstrated that FGFR4 was expressed in all cell lines examined (Fig. 1C). Taken together, FGF19 production was concomitant with FGFR4 expression in Huh7 and JHH7 cells (Fig. 1D).

### Loss-of-function assays of *FGFR4* in HCC cells

**Figure 2:**
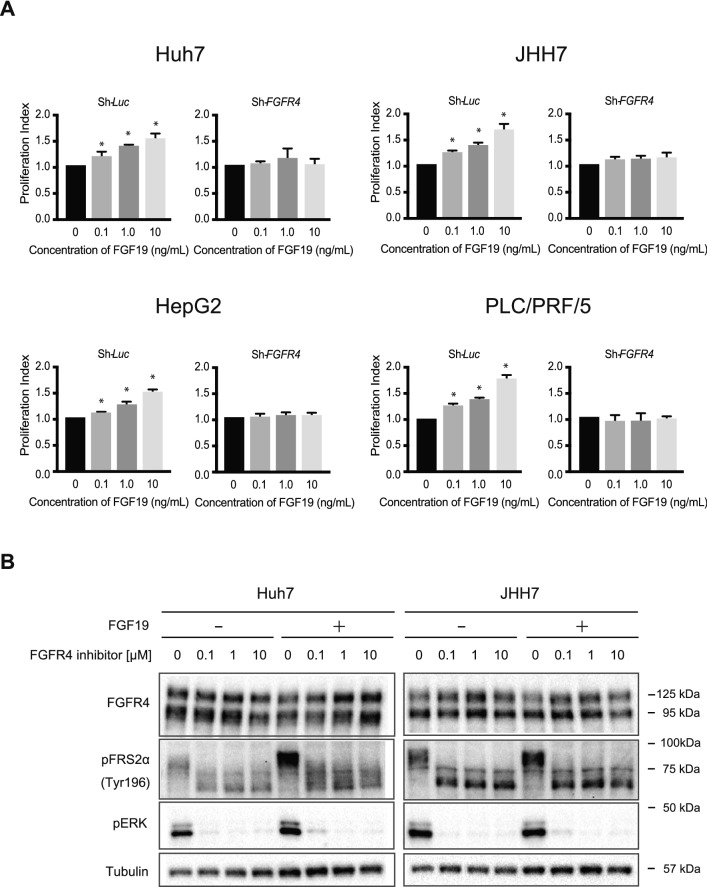
In vitro assays of FGF19-stimulated HCC cells. (**A**) Proliferation of HCC cells transduced with the indicated lentiviruses in culture 96 h after seeding. These data were from three independent experiments. *Significant (*p* < 0.05). (**B**) Cells treated with FGF19 and/or a FGFR4 inhibitor were subjected to Western blot analyses using anti-FGFR4, anti-pFRS2α, anti-pERK and anti-tubulin (loading control) antibodies.

We next conducted *FGFR4*-knockdown assay using short hairpin RNA (shRNA) for *FGFR4*. shRNA targeting *luciferase* (*Luc*) was used as a control. Two different shRNAs, sh-*FGFR4*-1 and sh-*FGFR4*-2, markedly repressed FGFR4 protein expression (Fig. 1E). Although both shRNAs markedly repressed the growth of all cell lines examined, this repression was more evident in FGF19^+^/FGFR4^+^ cell lines (Huh7 and JHH7 cells, Fig. 1F). We then examined the effects of recombinant FGF19 treatment in control and *FGFR4*-knockdown HCC cells. FGF19 altered proliferation in dose-dependent manner in control cells (Fig. 2A). As expected, FGF19-induced cell growth was lost in *FGFR4*-knockdown HCC cells. These results suggested that FGF19/FGFR4 signaling is closely associated with the cell growth activity of HCC cells.

Next, the expression of the adapter protein of FGFR4 and downstream target of FGFR4 were examined in Huh7 and JHH7 cells treated with recombinant FGF19 and/or BLU-9931, a selective FGFR4 inhibitor. Western blotting analyses using phospho-FRS2α (pFRS2α) and phospho-ERK (pERK) antibodies revealed that phosphorylation of both proteins was prominently suppressed by a selective FGFR4 inhibitor in not only parental cells but also recombinant FGF19-treated cells (Fig. 2B).

### Combined use of a selective FGFR4 inhibitor and MKIs

**Figure 3:**
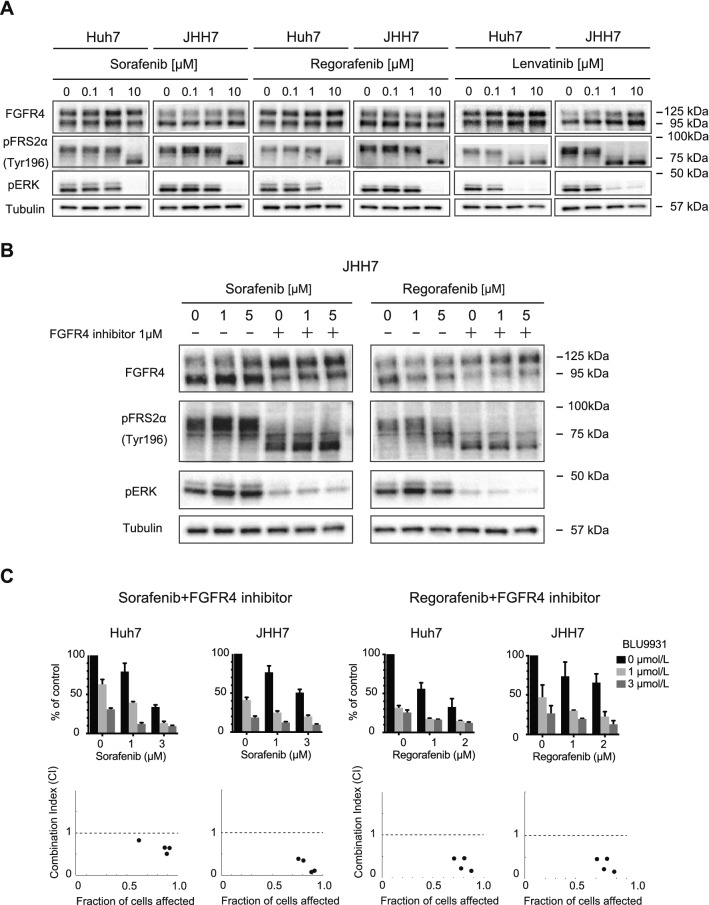
FGFR4/ERK signaling inhibition in HCC cells treated with MKIs and a FGFR4 selective inhibitor. (**A**, **B**) Cells treated with MKIs (**A**), and co-treated with MKIs and BLU-9931, a FGFR4 selective inhibitor (**B**), were subjected to Western blot analyses using anti-FGFR4, anti-pFRS2α, anti-pERK and anti-tubulin (loading control) antibodies. (**C**) Synergistic inhibitory effects of MKIs and FGFR4 selective inhibitor on HCC cell proliferation 120 h after seeding. These data were from three independent experiments. The combination index (CI) is shown below each graph.

To examine the effects of MKIs on pFRS2α and pERK levels, Western blotting was performed in HCC cells treated with MKIs at varying concentrations (Fig. 3A). Decreases in pFRS2α and pERK levels were observed in HCC cells treated with MKIs in a dose-dependent manner. Although 10 μM sorafenib and regorafenib treatment resulted in FGFR4 signaling inhibition, only 1 μM lenvatinib was sufficient. Cotreatment of a selective FGFR4 inhibitor (1 μM) and sorafenib or regorafenib successfully inhibited pFRS2α and pERK expression (Fig. 3B). Next, we examined the effects of the combined use of a selective FGFR4 inhibitor and MKIs (sorafenib or regorafenib) on cell growth activity. Additional treatment of a selective FGFR4 inhibitor enhanced cell growth inhibition followed by sorafenib or regorafenib treatment (Fig. 3C). Of note, synergistic effects of a selective FGFR4 inhibitor and sorafenib or regorafenib on the proliferation of HCC cells were confirmed by calculating the combination index (CI < 1).

### MKI and/or selective FGFR4 inhibitor treatment in a xenograft transplantation model

**Figure 4:**
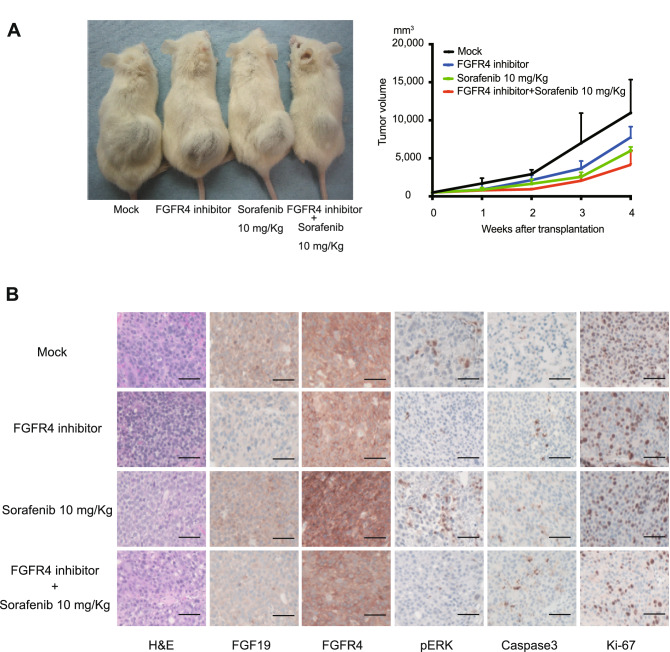
Xenograft transplantation and immunohistochemical analyses of NOD/SCID mice. (**A**) A total of 2 × 10^6^ Huh7 cells were transplanted into the subcutaneous areas of NOD/SCID mice. Representative images of recipient mice treated with FGFR4 inhibitor (30 mg/Kg) and/or sorafenib (10 mg/Kg) 4 weeks after transplantation (left panel). The tumor volume was monitored weekly after cell transplantation (right panel). (**B**) Hematoxylin and eosin (H&E) staining and immunohistochemical analyses of subcutaneous tumors. Scale bar = 100 μm.

To examine the anti-tumor effects of MKIs and a selective FGFR4 inhibitor in vivo, we conducted xenograft transplantation using Non-obese diabetic/severe combined immunodeficiency (NOD/SCID) mice. Sorafenib (10 mg/Kg) and/or a selective FGFR4 inhibitor (30 mg/Kg) was administered daily after the subcutaneous tumors reached 10 mm in diameter. Although tumor growth was obviously inhibited by the sorafenib and selective FGFR4 inhibitor treatment, it was further suppressed by the co-treatment of sorafenib and a selective FGFR4 inhibitor (Fig. 4A). Although the tumor volume in each group was not significantly different, there was a stronger tendency for tumor regression in the combination group than in the single agent group and in control group.

Next, subcutaneous tumors were subjected to immunohistochemical analyses. Of note, phosphorylation of ERK was inhibited by the administration of a FGFR4 inhibitor. Ki-67 and caspase3 immunostaining demonstrated that the co-treatment of sorafenib and selective FGFR4 inhibitor efficiently inhibited cell proliferation and induced apoptosis (Fig. 4B).

### Expression of FGF19/FGFR4 in primary HCC tissues

Although gene amplification of FGF19 is observed at 10–20% in HCC, the copy number variation and mRNA expression do not necessarily coincide^21^. Indeed, analyses of The Cancer Genome Atlas (TCGA) data revealed many cases of high FGF19 mRNA levels regardless of gene amplification (Supplementary Fig. S1). Therefore, in order to investigate the expression of FGF19, and the correlation between FGF19 and FGFR4, which is the main receptor for FGF19, 92 HCC tumor and adjacent non-tumor tissue samples were subjected to immunohistochemical analyses.

**Figure 5:**
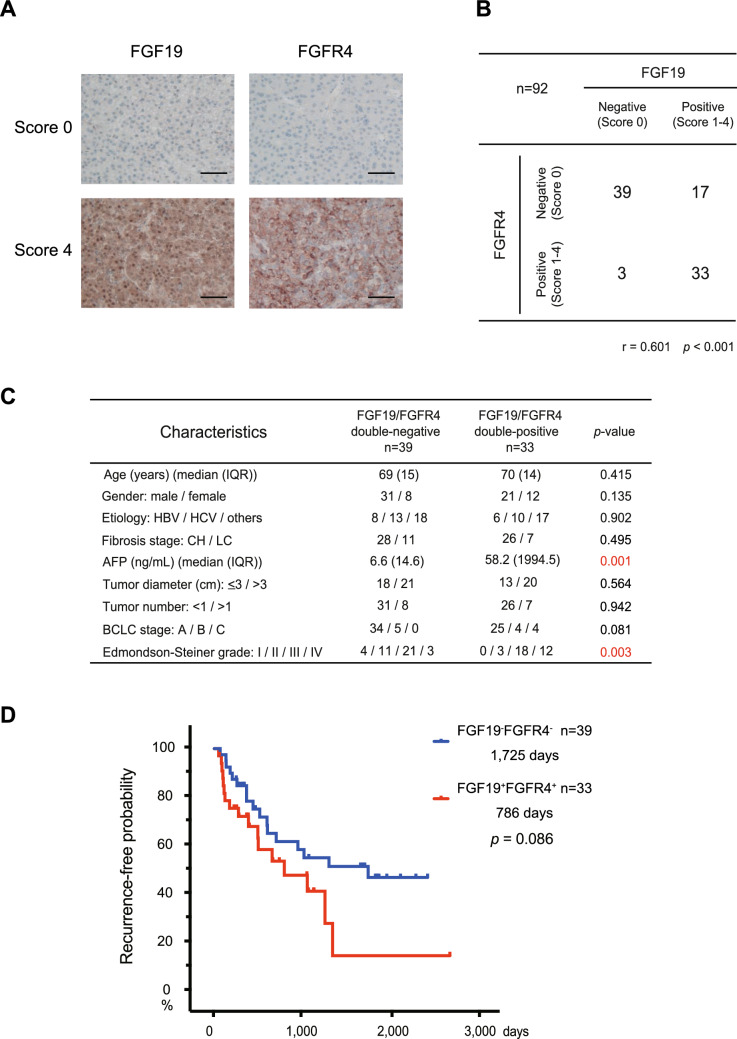
FGF19/FGFR4 expression in HCC surgical samples. (**A**) Representative FGF19/FGFR4 immunohistochemical staining of primary HCC tissues (score 0 or 4). Scale bar = 100 μm. (**B**) Correlation between FGF19 and FGFR4 expression. (**C**) Clinicopathological features of FGF19^+^FGFR4^+^ and FGF19^−^FGFR4^−^ HCC. (**D**) Cumulative RFS rate based on the FGF19/FGFR4 expression pattern.

Primary HCC tissues consist of tumor cells with varying expression of FGF19/FGFR4 (Fig. 5A). Based on FGF19 expression, the numbers of HCC samples scored as 0, 1, 2, 3 and 4 were 42 (45.7%), 17 (18.5%), 15 (16.3%), 13 (14.1%) and 5 (5.4%), respectively. Similarly, based on FGFR4 expression, the numbers of HCC samples scored as 0, 1, 2, 3 and 4 were 56 (60.9%), 12 (13.0%), 11 (12.0%), 6 (6.5%) and 7 (7.6%), respectively. To examine the correlation between the expression of FGF19/FGFR4 in tumor tissues, FGF19 and FGFR4 expression was classified as negative (score 0) or positive (scores 1–4), respectively (Fig. 5B). The expression of FGF19 was similar to FGFR4 in more than half of the cases, and a significant correlation was noted between the expression of FGF19 and FGFR4 in tumor tissues (r = 0.601, *p* < 0.001).

Focusing on the co-expression of FGF19/FGFR4, we compared the patient characteristics between FGF19^+^FGFR4^+^ HCC (n = 33) and FGF19^−^FGFR4^−^ HCC (n = 39) (Fig. 5C). Clinicopathological examination demonstrated that FGF19^+^FGFR4^+^ HCC was significantly associated with a high serum level of AFP (*p* = 0.001) and low differentiation pathology (*p* = 0.003). In addition, they were subjected to the prognostic analyses by the Kaplan‐Meier method. The presence of vascular invasion or tumor satellite nodules are well known factors implicated in recurrence^22^. In fact, the recurrence-free survival (RFS) in patients with vascular invasion or tumor satellite nodules was significantly shorter than that in patients without vascular invasion and tumor satellite nodules in this cohort (592 days vs. 1725 days, *p* = 0.006). In FGF19^+^FGFR4^+^ HCC and FGF19^−^FGFR4^−^ HCC groups, 39.4% and 23.1% of patients had vascular invasion or tumor satellite nodules, respectively. There was no statistically significant difference between FGF19^+^FGFR4^+^ HCC and FGF19^−^FGFR4^−^ HCC groups regarding vascular invasion or tumor satellite nodules (*p* = 0.134). Although there were no significant differences, the RFS in patients with FGF19^+^FGFR4^+^ HCC was slighter shorter than that in patients with FGF19^−^FGFR4^−^ HCC (*p* = 0.086, Fig. 5D).

### Potential of serum FGF19 as a marker for MKI treatment effects

**Figure 6:**
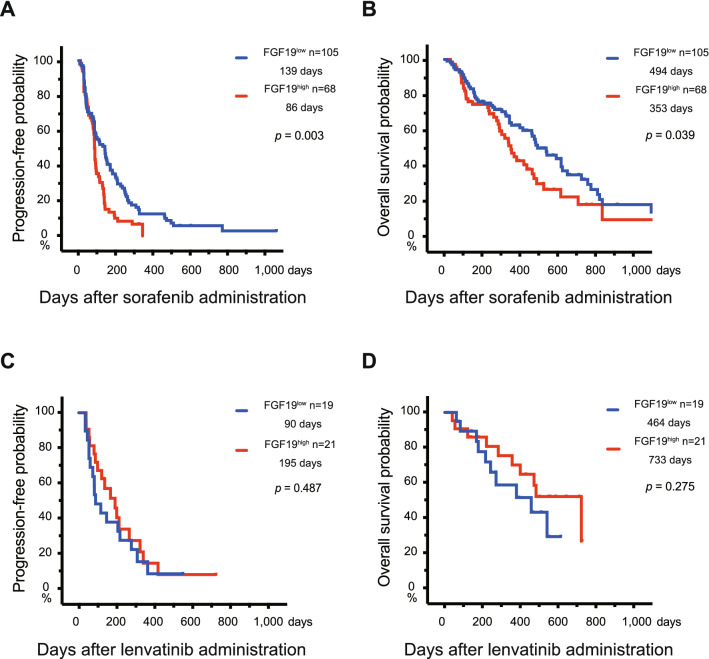
Prognosis of HCC patients treated using MKIs based on serum FGF19 levels before treatment. (**A**, **B**) Progression-free survival (PFS) (**A**) and overall survival (OS) (**B**) of patients treated using sorafenib based on serum FGF19 levels. (**C**, **D**) PFS (**C**) and OS (**D**) of patients treated using lenvatinib based on serum FGF19 levels.

Lastly, the prognosis of HCC patients treated using sorafenib or lenvatinib was assessed by the Kaplan–Meier method based on serum FGF19 levels before MKI treatment. The cut-off value of serum FGF19 (200 pg/mL) was used to divide HCC cases into 2 groups (FGF19^low^ and FGF19^high^ groups) according to our previous report^23^. There was no clinical feature difference between serum FGF19^high^ and FGF19^low^ patients treated using sorafenib and lenvatinib (Supplementary Tables S2 and S3). In sorafenib-administered cases, FGF19^low^ patients exhibited a significantly longer progression-free survival (PFS) and overall survival (OS) than FGF19^high^ patients (Fig. 6A, B). The median PFS in the FGF19^low^ and FGF19^high^ groups was 139 days and 86 days, respectively. Similarly, the median OS was 494 days and 353 days, respectively. In contrast, the PFS and OS of FGF19^high^ patients were modestly longer than those of FGF19^low^ patients in lenvatinib-administered cases, but there were no significant differences between two groups. The median PFS in the FGF19^low^ and FGF19^high^ groups was 90 days and 195 days, respectively. Similarly, the median OS was 464 days and 733 days, respectively (Fig. 6C, D).

## Discussion

Although driver gene mutations, such as in the TERT promoter, beta-catenin, and Tp53, are often observed in HCC, the frequency of mutations in other genes is not high^24,25^. As druggable gain-of-function mutations, such as in the epidermal growth factor receptor (EGFR) gene, are seldom detected, no therapeutic agents targeting a single gene mutation have been clinically applied^26^. Considering the genetic characteristics of HCC mentioned above, MKIs, inhibitors of multiple tyrosine kinase receptors, may be reasonable for HCC treatment. FGFR4 is one of the important targets for MKIs. FGF19/FGFR4 signaling functions as an essential cell growth signal in many solid tumors^27,28^. Together, inhibition of FGF19/FGFR4 signaling may play a role in the anti-tumor effects of MKIs against HCC. In this study, we investigated the impact of the regulation of FGF19/FGFR4 signaling on treatment using MKIs, namely sorafenib, regorafenib and lenvatinib.

To gain insight into the role of FGFR4, we conducted the loss-of-function assay of FGFR4 in culture. *FGFR4*-knockdown markedly suppressed cell growth in Huh7 and JHH7 cells (FGF19^+^FGFR4^+^ HCC cells), but not in HepG2 and PLC/PRF/5 cells (FGF19^−^FGFR4^+^ cells). In addition, recombinant FGF19 treatment increased cell proliferation in Huh7, JHH7, HepG2 and PLC/PRF/5 cells (FGF19^+^FGFR4^+^ HCC cells and FGF19^−^FGFR4^+^ cells). These results suggested that FGF19/FGFR4 signaling activity directly affects the proliferation of HCC cells. We next examined the effects of MKIs on FGFR4-mediated ERK signaling by Western blotting. Of note, lenvatinib markedly reduced pFRS2 and pERK levels compared with sorafenib and regorafenib. Furthermore, co-treatment of sorafenib or regorafenib and a selective FGFR4 inhibitor, BLU-9931 caused synergistic cell growth inhibition accompanied by reduced pFRS2 and pERK. Taken together, growth inhibition following lenvatinib treatment may be due to suppression of the FGFR4-mediated RAS/RAF/MAPK pathway.

Our analyses revealed that the combined use of sorafenib or regorafenib with a selective FGFR4 inhibitor suppressed FGFR4-mediated ERK signaling, thereby producing additional anti-tumor effects. A selective FGFR4 inhibitor, BLU-554, was previously applied in clinical trials with favorable outcomes in some HCC patients^29^. Pan-FGFR inhibitors have also been recently developed and applied in clinical studies on different cancer types^30–32^. As FGFR1 activation also function in sorafenib resistance^33^, co-treatment of sorafenib or regorafenib and pan-FGFR inhibitors may be useful.

FGF19 gene amplification was reported in 10–20% of HCC cases. However, in the present analyses utilizing TCGA data, FGF19 amplification was not correlated with its RNA expression. Few genes with an increased copy number have an increased protein level^34^. Our clinicopathological examination revealed that FGF19 and FGFR4 were concomitantly expressed in approximately one-third of the HCC tissue samples. FGF19^+^FGFR4^+^ HCC is associated with biological malignancy, represented by low differentiation pathology and unfavorable prognosis. Together, these results suggest that FGF19/FGFR4 signaling is activated in some HCC cases and this may be closely related to progression.

Biomarkers are utilized in many aspects of medical treatment for cancer patients such as early diagnosis, recurrence diagnosis and prediction of prognosis^35^. Among them, predictive biomarkers for treatment outcome are important in HCC patients. Ideally, they should be functional, and evaluated before drug selection and administration^36^. As FGF19 and FGFR4 exhibited similar expression pattern in HCC tissues, we considered the possibility that measurement of autocrine secretion of FGF19 in HCC patients sera reflects the dependency upon FGF19/FGFR4 signaling. As expected, high serum FGF19 levels were associated with an unfavorable OS and PFS in patients treated using sorafenib, but not in patients treated using lenvatinib. Taking into consideration the marked inhibitory effects of lenvatinib on FGF19/FGFR4 signaling in culture compared with sorafenib, these findings are reasonable.

HCC is one of the typical hypervascular tumors, and angiogenesis plays an essential role in its development and progression^37^. MKIs inhibit both cell growth and tumor angiogenesis through the suppression of angiogenic receptor tyrosine kinases such as FGFR, vascular endothelial growth factor (VEGF) receptor (VEGFR) and platelet-derived growth factor receptor^38^. Lenvatinib has been reported to have excellent anti-tumor and anti-angiogenic effects in VEGF-overexpressing HCC xenograft models^39^. Further analyses are necessary to clarify whether these angiogenic markers or their combined use with FGF19 can also function as the markers for lenvatinib treatment response.

Our study demonstrated that robust inhibition of FGF19/FGFR4 is of importance for the exertion of anti-tumor effects by MKIs. In addition, serum FGF19 levels may act as a predictive marker for drug response and survival in HCC patients treated using MKIs.

## Materials and methods

### Cell culture and reagents

Human HCC cell lines (Huh7, JHH7, HepG2 and PLC/PRF/5 cells) were obtained from the Health Science Research Resources Bank (HSRRB, Osaka, Japan). Cells were cultured in Dulbecco’s modified Eagle’s medium (Invitrogen Life Technologies, Carlsbad, CA, USA) supplemented with 10% fetal calf serum and 1% penicillin/streptomycin (Invitrogen). Recombinant human FGF19 was obtained from PeproTech Inc. (Rocky Hill, NJ, USA). Sorafenib was purchased from LKT laboratories (Saint Paul, MN, USA), and regorafenib and BLU-9931 were purchased from Cayman Chemicals (Ann Arbor, MI, USA). Lenvatinib was provided by Eisai Inc. (Tokyo, Japan).

### ELISA

Concentrations of FGF19 in culture supernatant and serum FGF19 in HCC patients were measured using a sandwich ELISA according to the manufacturer’s instructions (R&D Systems, Inc., Minneapolis, MN, USA). FGF19 production ability of an HCC cell line was assessed using culture supernatant collected 24 h after plating 100,000 cells or 500,000 cells. The serum FGF19 concentration in HCC patients was measured before the administration of MKIs.

### Western blotting

Parental HCC cells and MKI-treated cells were subjected to Western blot analyses with anti-FGFR4 (Cell Signaling Technology), anti-phospho-FRS2α (Tyr196, Cell Signaling Technology), anti-phospho-ERK1/2 (Thr202/Tyr204, Cell Signaling Technology) and anti-tubulin (Oncogene Science, Cambridge, MA, USA) antibodies.

### Lentiviral production and transduction

Lentiviral vectors (CS-H1-shRNA-EF-1a-EGFP) expressing shRNA targeting human *FGFR4* (target sequence: sh-*FGFR4*-1, 5′-GCAGAATCTCACCTTGATTAC-3′; sh-*FGFR4*-2, 5′-GCGTCCACCACATTGACTACT-3′) and *Luc* were constructed. Recombinant lentiviruses were produced as described previously^40^. Cells were transduced with a lentiviral vector in the presence of protamine sulfate (10 μg/mL; Sigma, St. Louis, MO, USA).

### Trypan blue dye exclusion test and calculation of the combination index

Cell growth of HCC cells was assessed by trypan blue staining after 60, 96 or 120 h in culture. The proliferation index (PI) was defined as the cell number of the recombinant human FGF19-treated cells divided by those of the untreated control cells. The combined effects of a selective FGFR4 inhibitor with sorafenib or regorafenib was analyzed by isobologram analyses using the CompuSyn software version 1.0 (http://www.combosyn.com/, ComboSyn, Inc, Paramus, NJ, USA)^41–43^.

### Xenograft transplantation

NOD/SCID mice (Sankyo Laboratory Co. Ltd., Tsukuba, Japan) were bred and maintained according to our institutional guidelines for the use of laboratory animals. A total of 2 × 10^6^ Huh7 cells were suspended in 200 uL of DMEM and Matrigel (BD Biosciences, Bedford, MA, USA) (1:1), and implanted into the subcutaneous area on the mice back. Sorafenib (10 mg/Kg) and BLU-9931 (30 mg/Kg) were administered daily by oral gavage. Subcutaneous tumors were subjected to hematoxylin and eosin (H&E) staining and immunohistochemistry with anti-FGF19 (Atlas antibodies, Bromma, Sweden), anti-FGFR4, anti-phospho-ERK, anti-CASP3 (Millipore, Billerica, MA, USA) and anti-Ki67 (DAKO, Carpinteria, CA, USA) antibodies. These experiments were performed according to both the institutional guidelines for the use of laboratory animals and the ARRIVE guidelines (https://arriveguidelines.org).

### Patients and surgical specimens

A total of 92 pairs of tumor and adjacent non-tumor tissue were pathologically analyzed. All patients provided informed consent. Paraffin-embedded tumor sections and the surrounding non-tumor tissues were examined by H&E staining and immunohistochemistry with anti-FGF19 and anti-FGFR4 antibodies. Based on the percentage of HCC cells positive for FGF19 or FGFR4, HCC tissues were classified as: no staining (score 0); 1–25% of cells (score 1); 26–50% of cells (score 2); 51–75% of cells (score 3) or more than 76% of cells (score 4). All patients received postoperative radiological follow-up every 2–6 months. Radiological assessments were evaluated according to the Response Evaluation Criteria in Solid Tumors^44^. This study was approved by the research ethics committees of the Graduate School of Medicine, Chiba University (approval number: 3300).

### Patients and blood samples

Blood samples were collected from 173 and 40 patients treated using sorafenib and lenvatinib, respectively, for HCC at the Chiba University Hospital between June 2012 and July 2019. All patients had not previously received systemic treatments. Serum samples were collected before treatment initiation. After obtaining informed consent, we analyzed the preserved blood samples and data from their medical records. Radiological assessments were evaluated according to the Response Evaluation Criteria in Solid Tumors. This study was approved by the research ethics committees of the Graduate School of Medicine, Chiba University (approval number: 3024).

### Data collection and analysis from TCGA–Liver Hepatocellular Carcinoma (LIHC)

RNA sequencing dataset (ID: TCGA.LIHC.sampleMap/HiSeqV2) and whole genome microarray dataset (ID: TCGA.LIHC.sampleMap/Gistic2_CopyNumber_Gistic2_all_thresholded.by_genes) were downloaded using the UCSC Xena Browser (https://xenabrowser.net/). In those datasets, the two classes of phenotypes were named “primary tumor” and “solid tissue normal”, only those samples in the clinical category of “primary tumor” were for this study. RNA sequencing dataset shows the gene-level transcription estimates, as in log2(x + 1) transformed RSEM normalized count. Whole genome microarray dataset shows the gene-level copy number estimates values, as in − 2, − 1, 0, 1, 2 using GISTIC2 method. We defined − 2, − 1 and 0 as amplification negative and 1 and 2 as amplification positive. Subsequently, Both FGF19 expression and its amplification in HCC were subjected to secondary analyses.

### Statistical analysis

Data are presented as the mean with standard deviation (SD), or the median with minimum to maximum and interquartile range (IQR). Statistical differences in the quantitative values between groups were determined by either Student’s *t* test or the Mann–Whitney *U* test. Chi-square test was used for categorical values. The relationship between FGF19 and FGFR4 expression based on immunohistochemical staining was analyzed by the Kendall rank correlation coefficient. The log-rank test was used to analyze survival data. The level of significance was set as *p* < 0.05. All statistical analyses were performed using the SPSS statistical software version 24 (IBM, Chicago, IL, USA).

## Supplementary Information

Supplementary Information

## References

[1] Bray F (2018). Global cancer statistics 2018: GLOBOCAN estimates of incidence and mortality worldwide for 36 cancers in 185 countries. CA Cancer J. Clin..

[2] Llovet JM, Burroughs A, Bruix J (2003). Hepatocellular carcinoma. Lancet.

[3] Jemal A (2011). Global cancer statistics. Cancer J. Clin..

[4] Kawano Y (2008). Short- and long-term outcomes after hepatic resection for hepatocellular carcinoma with concomitant esophageal varices in patients with cirrhosis. Ann. Surg. Oncol..

[5] Stefaniuk P, Cianciara J, Wiercinska-Drapalo A (2010). Present and future possibilities for early diagnosis of hepatocellular carcinoma. World J. Gastroenterol..

[6] Shibata T, Aburatani H (2014). Exploration of liver cancer genomes. Nat. Rev. Gastroenterol. Hepatol..

[7] Llovet JM, Villanueva A, Lachenmayer A, Finn RS (2015). Advances in targeted therapies for hepatocellular carcinoma in the genomic era. Nat. Rev. Clin. Oncol..

[8] Llovet JM (2008). Sorafenib in advanced hepatocellular carcinoma. N. Engl. J. Med..

[9] Bruix J (2017). Regorafenib for patients with hepatocellular carcinoma who progressed on sorafenib treatment (RESORCE): A randomised, double-blind, placebo- controlled, phase 3 trial. Lancet.

[10] Kudo M (2018). Lenvatinib versus sorafenib in first-line treatment of patients with unresectable hepatocellular carcinoma: A randomised phase 3 non-inferiority trial. Lancet.

[11] Kudo M (2018). Lenvatinib May Drastically Change the Treatment Landscape of Hepatocellular Carcinoma. Liver Cancer..

[12] Kudo M (2018). Cabozantinib as a second-line agent in advanced hepatocellular carcinoma. Liver Cancer..

[13] Zhang F (2015). Minireview: Roles of fibroblast growth factors 19 and 21 in metabolic regulation and chronic diseases. Mol. Endocrinol..

[14] Katoh M, Nakagama H (2014). FGF receptors: Cancer biology and therapeutics. Med. Res. Rev..

[15] Eswarakumar VP, Lax I, Schlessinger J (2005). Cellular signaling by fibroblast growth factor receptors. Cytokine Growth Factor Rev..

[16] Kir S, Kliewer SA, Mangelsdorf DJ (2011). Roles of FGF19 in liver metabolism. Cold Spring Harb. Symp. Quant. Biol..

[17] Potthoff MJ, Kliewer SA, Mangelsdorf DJ (2012). Endocrine fibroblast growth factors 15/19 and 21: From feast to famine. Genes Dev..

[18] Lin BC, Desnoyers LR (2012). FGF19 and cancer. Adv. Exp. Med. Biol..

[19] Desnoyers LR (2008). Targeting FGF19 inhibits tumor growth in colon cancer xenograft and FGF19 transgenic hepatocellular carcinoma models. Oncogene.

[20] French DM (2012). Targeting FGFR4 inhibits hepatocellular carcinoma in preclinical mouse models. PLoS ONE.

[21] Caruso S (2019). Analysis of liver cancer cell lines identifies agents with likely efficacy against hepatocellular carcinoma and markers of response. Gastroenterology.

[22] Llovet JM, Schwartz M, Mazzaferro V (2005). Resection and liver transplantation for hepatocellular carcinoma. Semin. Liver Dis..

[23] Maeda T (2019). Serum fibroblast growth factor 19 serves as a potential novel biomarker for hepatocellular carcinoma. BMC Cancer..

[24] Totoki Y (2014). Trans-ancestry mutational landscape of hepatocellular carcinoma genomes. Nat. Genet..

[25] Schulze K (2015). Exome sequencing of hepatocellular carcinomas identifies new mutational signatures and potential therapeutic targets. Nat. Genet..

[26] Paez JG (2004). EGFR mutations in lung cancer: correlation with clinical response to gefitinib therapy. Science.

[27] Zhao X (2018). FGFR4 provides the conduit to facilitate FGF19 signaling in breast cancer progression. Mol. Carcinog..

[28] Turkington RC (2014). Fibroblast growth factor receptor 4 (FGFR4): A targetable regulator of drug resistance in colorectal cancer. Cell Death Dis..

[29] Kim R (2019). First-in-human phase I study of fisogatinib (BLU-554) validates aberrant FGF19 signaling as a driver event in hepatocellular carcinoma. Cancer Discov..

[30] Nogova L (2017). Evaluation of BGJ398, a fibroblast growth factor receptor 1–3 kinase inhibitor, in patients with advanced solid tumors harboring genetic alterations in fibroblast growth factor receptors: results of a global phase i, dose-escalation and dose-expansion study. J. Clin. Oncol..

[31] Michael M (2017). A phase 1 study of LY2874455, an oral selective pan-fgfr inhibitor, patients with advanced cancer. Target Oncol..

[32] Papadopoulos KP (2017). A Phase 1 study of ARQ 087, an oral pan-FGFR inhibitor in patients with advanced solid tumours. Br. J. Cancer..

[33] Tovar V (2017). Tumour initiating cells and IGF/FGF signalling contribute to sorafenib resistance in hepatocellular carcinoma. Gut.

[34] Zhang B (2014). Proteogenomic characterization of human colon and rectal cancer. Nature.

[35] Schrohl AS (2003). Tumor markers: From laboratory to clinical utility. Mol. Cell Proteomics..

[36] Ludwig JA, Weinstein JN (2005). Biomarkers in cancer staging, prognosis and treatment selection. Nat. Rev. Cancer..

[37] Zhu AX, Duda DG, Sahani DV, Jain RK (2011). HCC and angiogenesis: Possible targets and future directions. Nat. Rev. Clin. Oncol..

[38] Hung H (2010). Molecularly targeted therapy in hepatocellular carcinoma. Biochem. Pharmacol..

[39] Matsuki M (2018). Lenvatinib inhibits angiogenesis and tumor fibroblast growth factor signaling pathways in human hepatocellular carcinoma models. Cancer Med..

[40] Chiba T (2008). The polycomb gene product BMI1 contributes to the maintenance of tumor-initiating side population cells in hepatocellular carcinoma. Cancer Res..

[41] Chou TC, Talalay P (1984). Quantitative analysis of dose-effect relationships: The combined effects of multiple drugs or enzyme inhibitors. Adv. Enzyme Regul..

[42] Chou TC (2006). Theoretical basis, experimental design, and computerized simulation of synergism and antagonism in drug combination studies. Pharmacol. Rev..

[43] Chou TC (2010). Drug combination studies and their synergy quantification using the Chou-Talalay method. Cancer Res..

[44] Eisenhauer EA (2009). New response evaluation criteria in solid tumours: revised RECIST guideline (version 11). Eur. J. Cancer..

