# Supplementary material for: The impact of FGF19/FGFR4 signaling inhibition in antitumor activity of multi-kinase inhibitors in hepatocellular carcinoma
Source: Sci Rep. 2021 Mar 5;11:5303. doi: 10.1038/s41598-021-84117-9 (PMC7935880; doi:10.1038/s41598-021-84117-9)
Supplement: Supplementary file 1 — Supplementary Information [file 41598_2021_84117_MOESM1_ESM.pdf]

The impact of FGF19/FGFR4 signaling inhibition in antitumor activity of multi-kinase inhibitors in hepatocellular carcinoma

Authors:

Hiroaki Kanzaki[1], Tetsuhiro Chiba[1, *], Junjie Ao[1], Keisuke Koroki[1], Kengo Kanayama[1], Susumu Maruta[1], Takahiro Maeda[1], Yuko Kusakabe[1], Kazufumi Kobayashi[1], Naoya Kanogawa[1], Soichiro Kiyono[1], Masato Nakamura[1], Takayuki Kondo[1], Tomoko Saito[1], Ryo Nakagawa[1], Sadahisa Ogasawara[1], Eiichiro Suzuki[1], Yoshihiko Ooka[1], Ryosuke Muroyama[2], Shingo Nakamoto[1], Shin Yasui[1], Akinobu Tawada[1], Makoto Arai[1], Tatsuo Kanda[3], Hitoshi Maruyama[4], Naoya Mimura[5], Jun Kato[1], Yoh Zen[6], Masayuki Ohtsuka[7], Atsushi Iwama[8], Naoya Kato[1]

Institutions:

[1] Department of Gastroenterology, Graduate School of Medicine, Chiba University, 1-8-1 Inohana, Chuo-ku, Chiba 260-8670, Japan

[2] Department of Molecular Virology, Graduate School of Medicine, Chiba University, 1-8-1 Inohana, Chuo-ku, Chiba 260-8670, Japan

[3] Department of Gastroenterology and Hepatology, Nihon University School of Medicine, 30-1 Oyaguchi-Kamicho, Itabashi-ku, Tokyo 173-8610, Japan

[4] Department of Gastroenterology, Juntendo University School of Medicine, 2-1-1 Hongo, Bunkyo-ku, Tokyo 113-8421, Japan

[5] Department of Transfusion Medicine and Cell Therapy, Chiba University Hospital, 1-8-1 Inohana, Chuo-ku, Chiba 260-8670, Japan

[6] Institute of Liver Studies, King's College Hospital, London, UK

[7] Department of General Surgery, Graduate School of Medicine, Chiba University, 1-8-1 Inohana, Chuo-ku, Chiba 260-8670, Japan

[8] Division of Stem Cell and Molecular Medicine, Center for Stem Cell Biology and Regenerative Medicine, The Institute of Medical Science, The University of Tokyo, 4-6-1 Shirokanedai, Minato-ku, Tokyo 108-8639, Japan

***Corresponding author:**

Tetsuhiro Chiba, M.D., PhD.

Department of Gastroenterology, Graduate School of Medicine, Chiba University, 1-8-1 Inohana, Chuo-ku, Chiba 260-8670, Japan.

Telephone: +81-43-2262083, Fax: +81-43-2262088

E-mail: chibat@chiba-u.jp

Supplementary Table S1. Biochemical IC50 values (nmol/L) of MKIs.

	**Sorafenib**	**Lenvatinib**	**Regorafenib**	**Cabozantinib**
VEGFR-1	21	4.7	13	NA
VEGFR-2	21	3	4.2	14
VEGFR-3	16	2.3	46	NA
FGFR1	340	61	202	NA
FGFR2	150	27	NA	NA
FGFR3	340	52	NA	NA
FGFR4	3400	43	NA	NA
PDGFRα	1.6	29	NA	NA
PDGFRβ	27	160	22	575
c-KIT	140	85	7	752
RET	15	6.4	1.5	8
MET	NA	NA	NA	2
AXL	NA	NA	NA	8

Abbreviations, NA: not applicable

Supplementary Table S2. Clinical features of serum FGF19^{high} and FGF19^{low} patients treated using sorafenib.

Characteristics	FGF19^{low} (n=105)	FGF19^{high} (n=68)	*p*-value
Age (years) (median (IQR))	72 (12)	74 (12)	0.104
Gender: male / female	81/24	56/12	0.410
Etiology: HBV /HCV / others	16/51/38	13/26/29	0.407
Child-Pugh grade: A / B	87/18	54/14	0.569
FGF19 (pg/ml) (median (IQR))	111.4 (65.1)	325.0 (298.4)	<0.001[a]
AFP (ng/ml) (median (IQR))	254.2 (26629.5)	87.3 (2236.4)	0.528
Tumor diameter (cm): >6 / ≤6	34/71	21/47	0.836
Tumor number: ≥7 / <7	53/52	36/32	0.751
Macrovascular invasion: yes / no	34/71	22/46	0.997
Extrahepatic metastasis: yes / no	39/66	21/47	0.398
BCLC stage: A / B / C	3/38/64	1/26/41	0.822

[a] Significant.

Supplementary Table S3. Clinical features of serum FGF19^{high} and FGF19^{low} patients treated using lenvatinib.

Characteristics	FGF19^{low} (n=19)	FGF19^{high} (n=21)	p-value
Age (years) (median (IQR))	73 (11)	72 (15)	0.936
Gender: male / female	14/5	16/5	0.855
Etiology: HBV /HCV / others	2/4/13	1/8/12	0.447
Child-Pugh grade: A / B	15/4	14/7	0.385
FGF19 (pg/ml) (median (IQR))	135.4 (50.9)	307.5 (143.3)	<0.001[a]
AFP (ng/ml) (median (IQR))	221.0 (966.4)	34.6 (122.9)	0.247
Tumor diameter (cm): >6 / ≤6	7/12	11/10	0.324
Tumor number: ≥7 / <7	11/8	10/11	0.516
Macrovascular invasion: yes / no	8/11	5/16	0.217
Extrahepatic metastasis: yes / no	7/12	4/17	0.208
BCLC stage: A / B / C	0/6/13	3/8/10	0.166

[a] Significant.

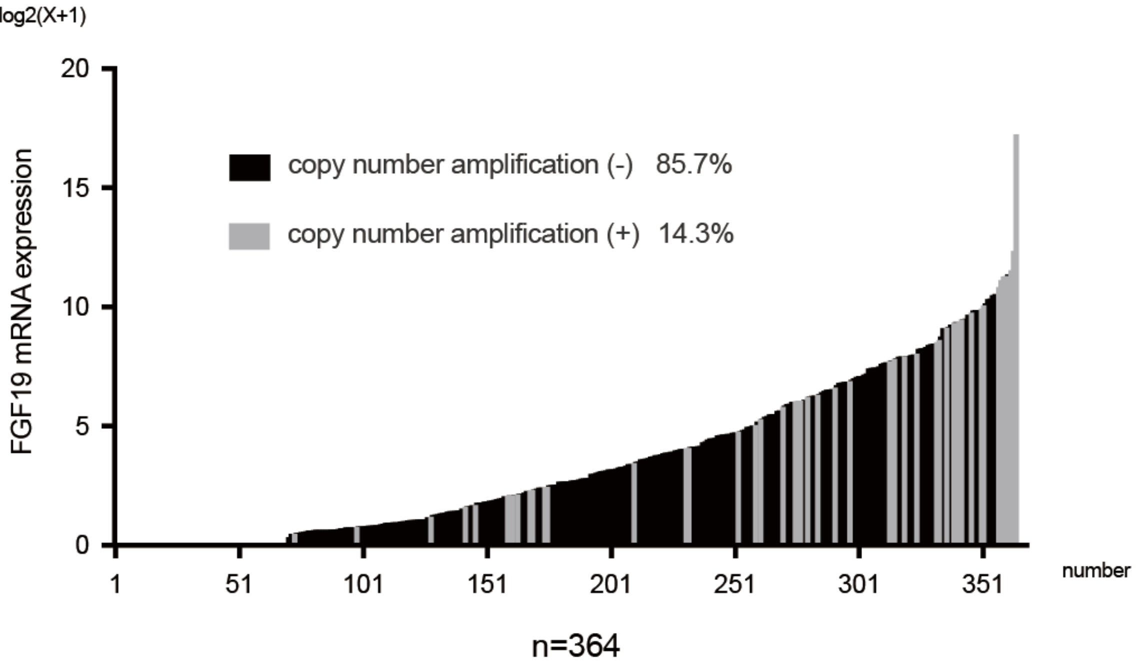

Supplementary Figure S1. Data collection and analysis of TCGA-LIHC. The correlation between the FGF19 mRNA level and FGF19 gene amplification in primary HCC (n=364) was depicted.

Figure 1C

FGFR4

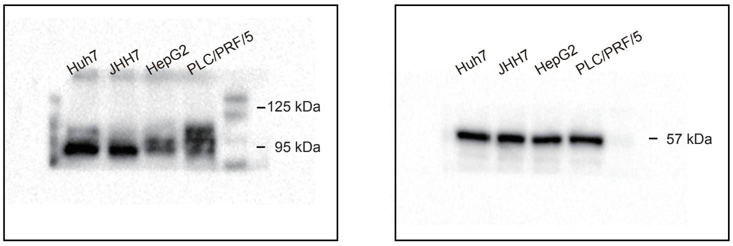

Tubulin

Figure 1E

FGFR4

Tubulin

FGFR4

Tubulin

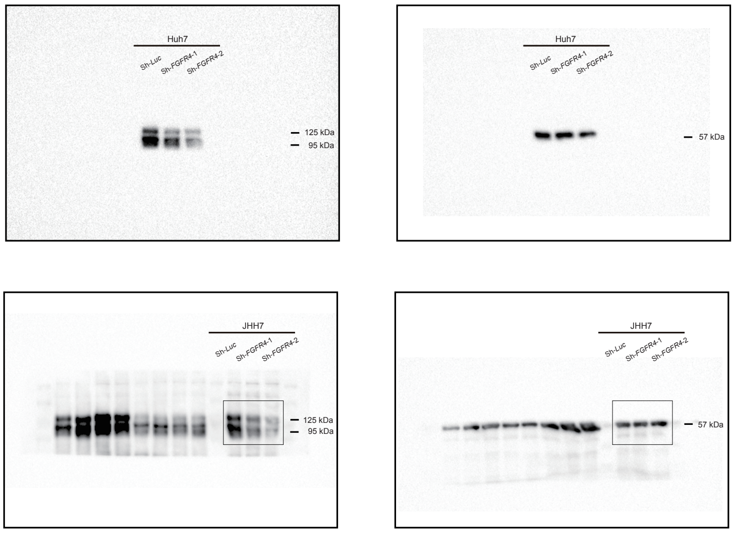

Supplementary Figure S2. Uncropped Western blot images of Figure 1C and Figure1E.

Figure 2B

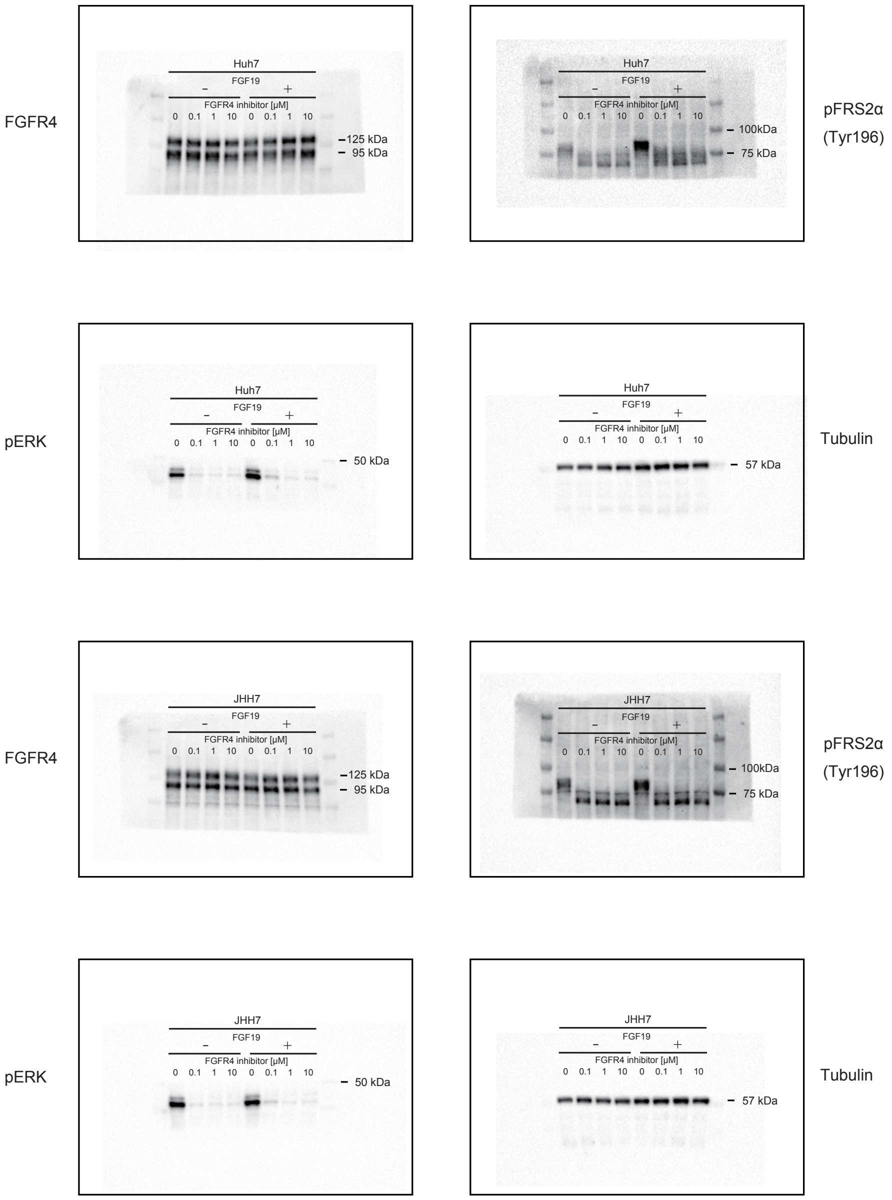

Supplementary Figure S3. Uncropped Western blot images of Figure 2B.

Figure 3A

FGFR4

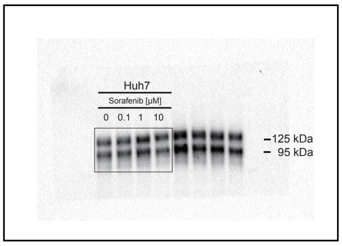

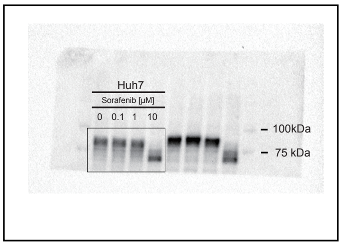

pFRS2α
(Tyr196)

pERK

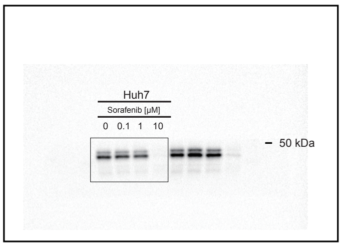

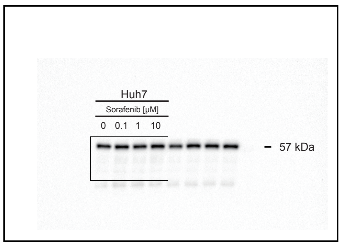

Tubulin

FGFR4

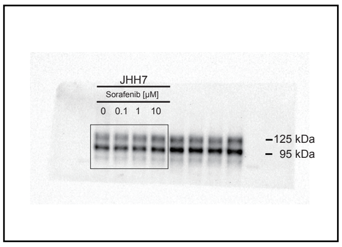

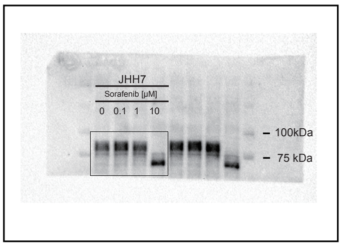

pFRS2α
(Tyr196)

pERK

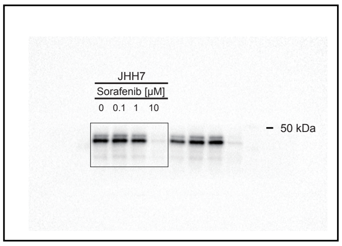

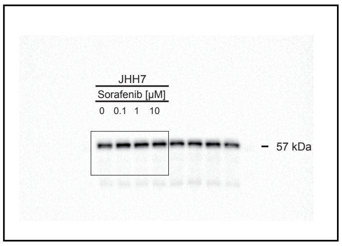

Tubulin

Supplementary Figure S4. Uncropped Western blot images of Figure 3A (left column).

Figure 3A

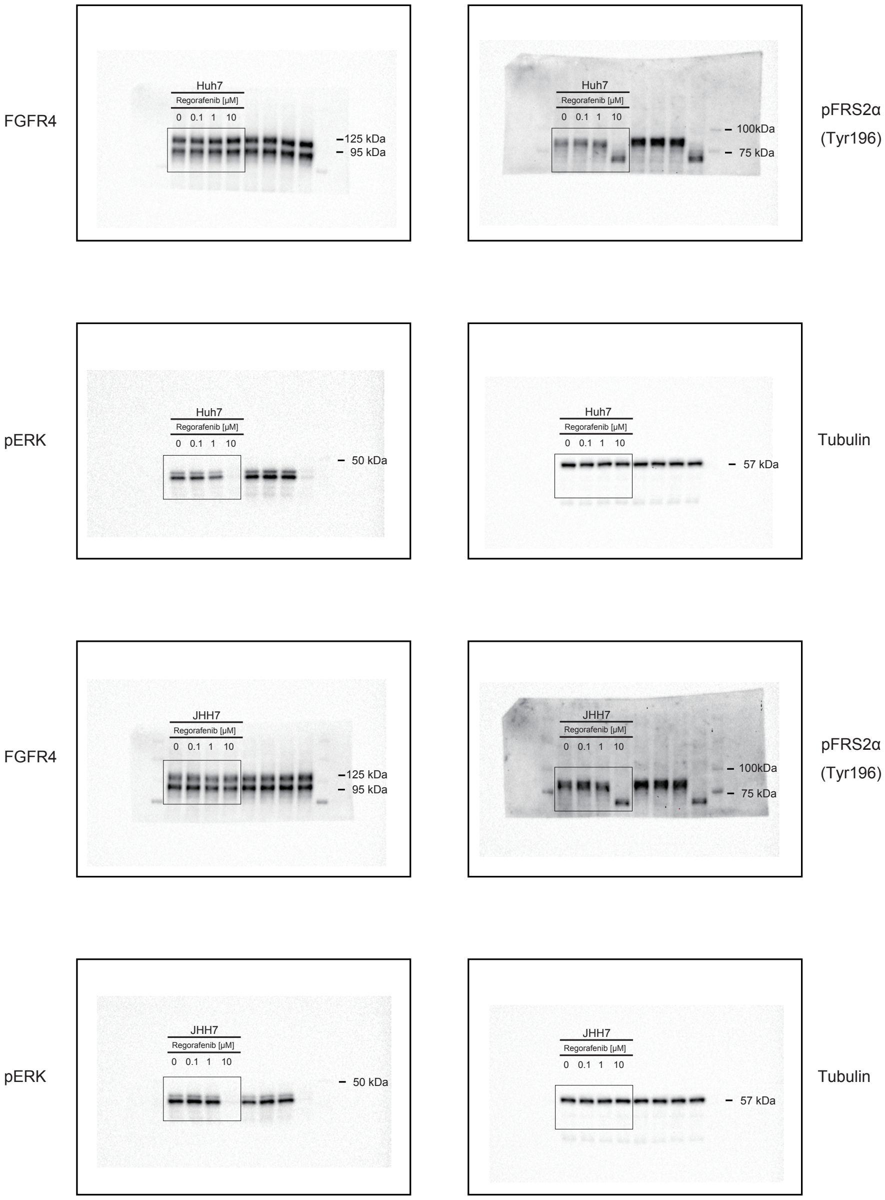

Supplementary Figure S5. Uncropped Western blot images of Figure 3A (middle column).

Figure 3A

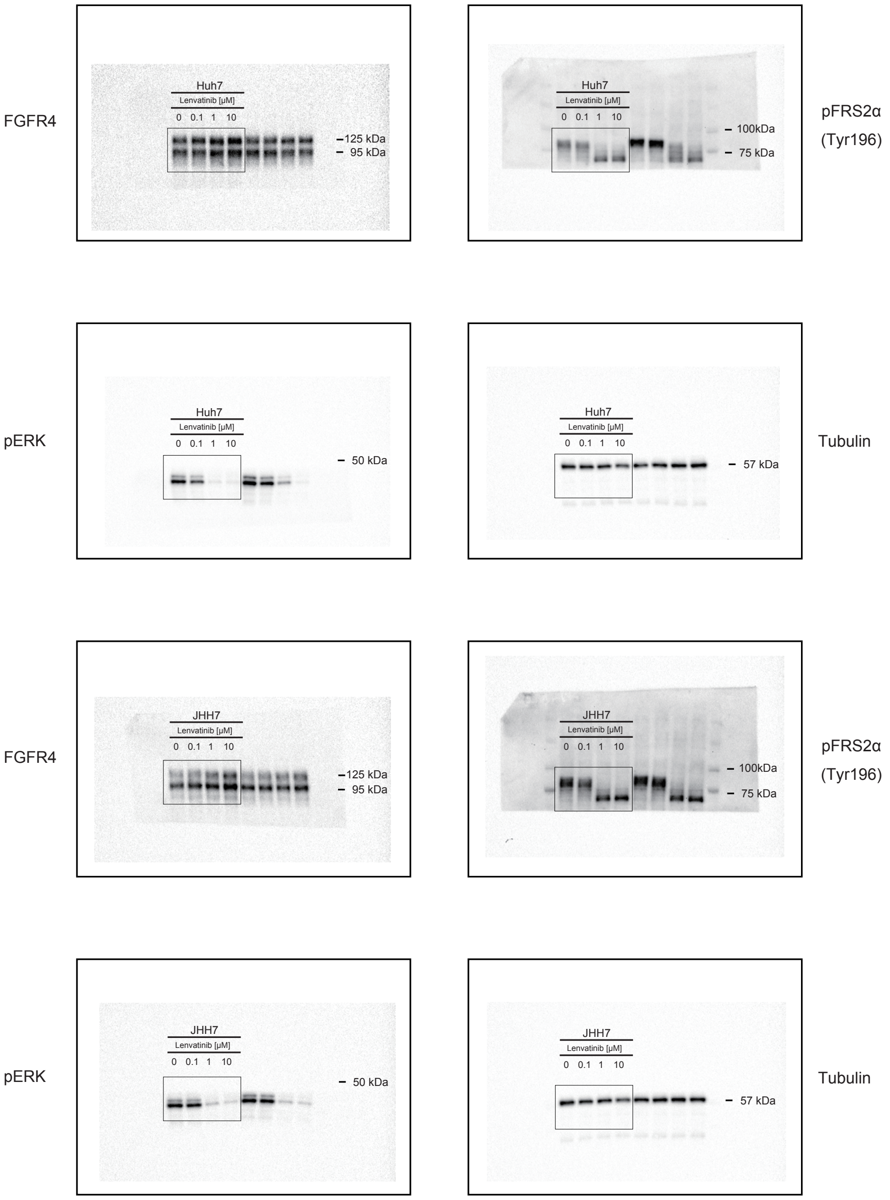

Supplementary Figure S6. Uncropped Western blot images of Figure 3A (right column).

Figure 3B

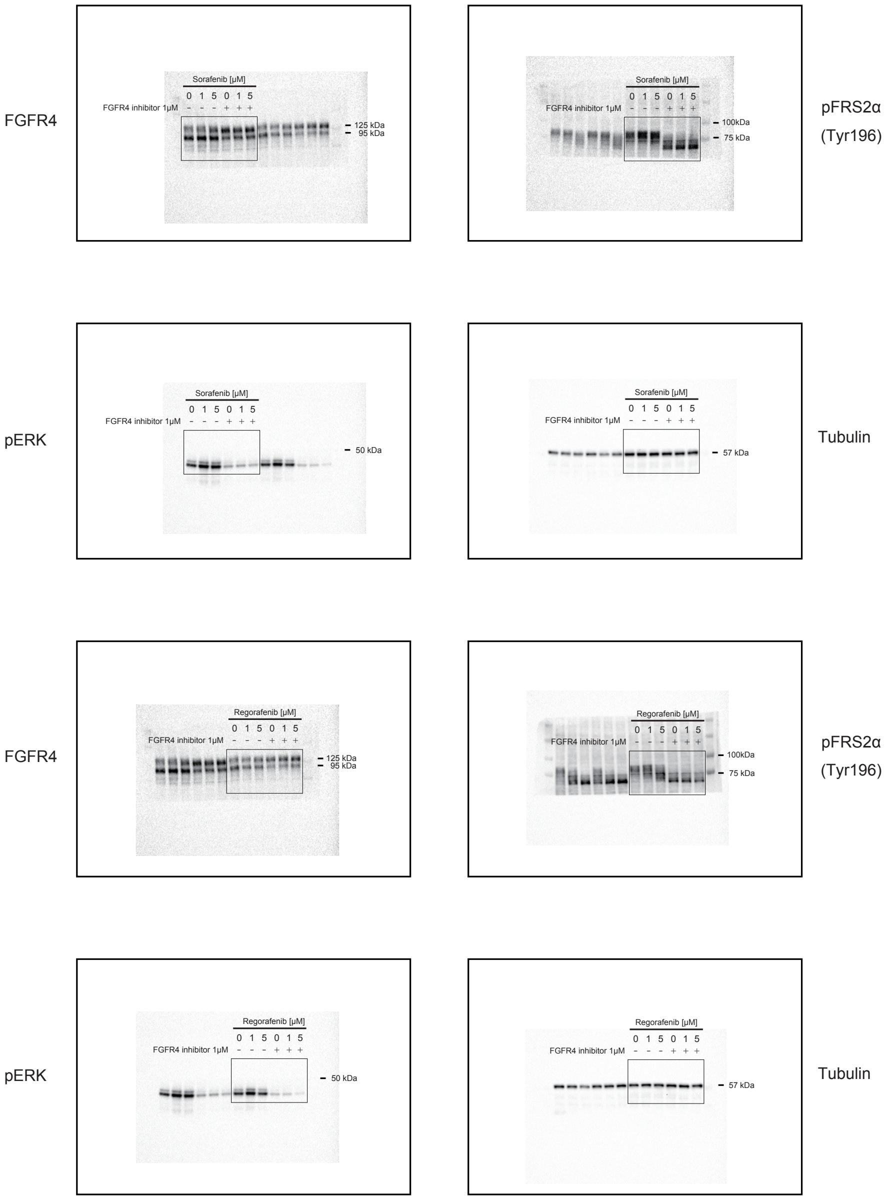

Supplementary Figure S7. Uncropped Western blot images of Figure 3B.
